# Biobased Polyurethane Composite Foams Reinforced with Plum Stones and Silanized Plum Stones

**DOI:** 10.3390/ijms22094757

**Published:** 2021-04-30

**Authors:** Karolina Miedzińska, Sylwia Członka, Anna Strąkowska, Krzysztof Strzelec

**Affiliations:** Faculty of Chemistry, Institute of Polymer & Dye Technology, Lodz University of Technology, 90-924 Lodz, Poland; sylwia.czlonka@dokt.p.lodz.pl (S.C.); anna.strakowska@p.lodz.pl (A.S.); krzysztof.strzelec@p.lodz.pl (K.S.)

**Keywords:** polyurethanes, reinforcement, plum stones, silanization, mechanical properties

## Abstract

In the following study, ground plum stones and silanized ground plum stones were used as natural fillers for novel polyurethane (PUR) composite foams. The impact of 1, 2, and 5 wt.% of fillers on the cellular structure, foaming parameters, and mechanical, thermomechanical, and thermal properties of produced foams were assessed. The results showed that the silanization process leads to acquiring fillers with a smoother surface compared to unmodified filler. The results also showed that the morphology of the obtained materials is affected by the type and content of filler. Moreover, the modified PUR foams showed improved properties. For example, compared with the reference foam (PUR_REF), the foam with the addition of 1 wt.% of unmodified plum filler showed better mechanical properties, such as higher compressive strength (~8% improvement) and better flexural strength (~6% improvement). The addition of silanized plum filler improved the thermal stability and hydrophobic character of PUR foams. This work shows the relationship between the mechanical, thermal, and application properties of the obtained PUR composites depending on the modification of the filler used during synthesis.

## 1. Introduction

In recent years, polymer composites have taken over the world market. The growing demand for polymer materials has increased the environmental requirements for these products. Therefore, scientists have begun to look for new methods of obtaining more natural additives for polymer composites consistent with the principles of Sustainable Development [1,2].

One of the groups of polymers with the most versatile properties is polyurethanes (PUR). The wide range of possibilities for manipulating the chemical components that make up the polymer backbone offers a huge variety of finished product characteristics and the ability to tailor their properties to specific applications [3,4]. Depending on the components and modifiers used in the synthesis, PUR materials with new and improved mechanical, chemical, physical, and functional properties can be obtained [5]. Polyurethanes are obtained by the formation of urethane bonds due to the reaction of polyols and polyisocyanates, discovered in 1937 by Bayer [6]. The use of properly selected polyols and polyisocyanates allows obtaining polyurethane materials in various forms, including elastomers, coatings, adhesives, rigid and flexible foams, films, and fibers [3,7,8]. As a result of such varieties in the microstructure and diversity properties of the obtained materials, polyurethanes have been widely used in many sectors, such as thermal and electrical insulation and coatings, biomedical applications, construction, high-performance adhesives, the footwear market, and packaging or furniture [9,10,11,12,13,14].

The main group of PUR materials are foams that dominate the market with about 65% of the production of polyurethane materials [6,15]. The main characteristic features of polyurethane foams are porous structure, low apparent density, and relatively high strength. Their individual properties can be adjusted through the appropriate selection of components and additives during synthesis [5,16]. Depending on the properties of the obtained porous materials, they are divided into two main groups: rigid polyurethane foams (RPUFs) and flexible polyurethane foams (FPUFs) [17]. The porous structure of PUR foams, especially the number of closed pores in the structure, determines two of the most important functional features, which are low thermal conductivity and good dimensional stability. The presence of closed cells in the structure hinders heat transfer and results in lower values of the thermal conductivity coefficient, which means better thermal insulation capacity than in the case of open-cell structure materials. The closed-cell structure is characteristic of rigid polyurethane foams, which are highly cross-linked materials [18]. As for the application, polyurethane foams are widely used in many industries, including refrigeration, automotive, furniture, and electronics [19]. Moreover, they are used as thermal and sound insulation materials and as low-cost materials in construction [6,20].

The growing environmental requirements, the need to reduce the amount of polymer waste, and the principles of Sustainable Development have influenced the research on modern modifiers obtained from natural, renewable sources [21]. In recent years, one of the most frequently used bioadditives in the process of obtaining polyurethanes are materials of plant origin, used both as biopolyols and biofillers [22,23,24,25,26,27]. Many researchers have investigated the use of various cellulosic and lignocellulosic bioadditives such as bamboo leaves [28], wood powder [29], fruit fibers and stones [30,31], nutshells [32,33], hemp and flax fibers [34], and many more. Natural raw materials from plants play a special role as bioadditives due to their low cost of obtaining and accessibility. The use of natural raw materials in the production of polymers will improve the management of this type of waste, which is important due to the increasing costs of storage and utilization [35]. What is interesting is in the case of the same fruit, the total amount of waste was calculated and is about 30% by weight. Fruit waste can be successfully incorporated into many different polymer matrices and make them more biodegradable [36].

Among the different types of fillers used as reinforcing filler for polyurethane foams, to date, there are no publications that were carried out to examine the enhancement of properties of polyurethane composite foams modified with the addition of *Prunus domestica* stones, commonly known as plum stones (PS). It is reported that over 400 different species are classified under *Prunus* [37]. Nowadays, plums are cultured in many parts of the world, including China, the United States, or Turkey. Plums may have been one of the first fruits domesticated by humans. Their remains have been found at archaeological sites exploring the Neolithic age [38,39]. Plums are fruits rich in phenolic compounds, characterized by high antioxidant activity, exceeding fruits such as apples, grapes, or bananas [40,41]. As described in the literature, plum seeds contain significant amounts of oil, containing various bioactive compounds, such as lipids, proteins, and phenolic compounds [42,43,44,45]. It has also been determined that among fatty acids, there are oleic, linoleic, stearic, palmitic, and arachidonic [46,47,48]. An ideal filler should have a positive effect on the modified (mechanical and functional) properties of the obtained materials. Plum filler is a lignocellulosic natural waste modifier, and by using this type of waste, the amount of natural waste could be reduced. Moreover, as described in the literature [28,29,30,31,32,33,34], the use of other lignocellulosic fillers positively influenced the properties of modified polymer materials. To the best of our knowledge, plum stones have not been used as a filler in polyurethane foams so far. The use of ground plum stones as a reinforcing filler can improve the physical and mechanical properties of PU composite foams.

Many natural fillers can be successfully used as reinforcing materials in polymers [49,50,51,52]. It was noticed that their hydrophilic character may limit their further application. Therefore, the surface modification of the used fillers seems to be an appropriate step before using natural fillers as reinforcing materials for polymer materials [53,54,55]. Previous studies have shown that chemical modifications of fillers’ surface can improve the adhesion and compatibility between the filler and the polymer matrix. Chemical modifications include alkalization [56], acetylation [57], benzoylation [58], and silanization [59]. Despite many published articles devoted to the research of polyurethane composites, no studies were found on the surface treatment of plum stones and the influence of silanized PS on the selected properties of the obtained PUR foams. Therefore, this study investigated the influence of unmodified and silanized PS on the morphological, mechanical, and thermal properties of PUR foams.

## 2. Results and Discussion

### 2.1. Filler Silanization and Production Process of PUR Composites

**Figure 1:**
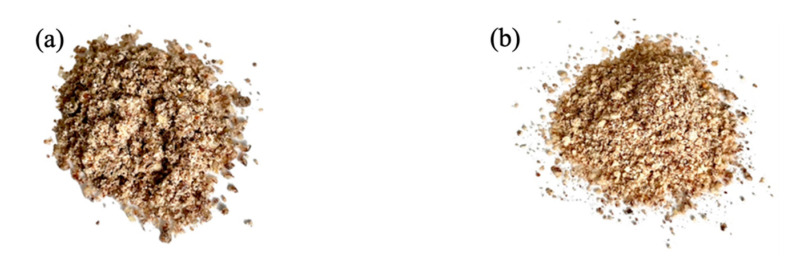
Optical image of (**a**) unmodified plum filler; (**b**) silanized plum filler.

Before application to the polyol system, the plum fillers were silanized, according to the following procedure. An ethanol/distilled water solution was prepared in a ratio of 80:20 (*v*/*v*%), then silane was added to obtain a 5% solution. The ground plum stones were added to the solution in a silane/filler ratio of 1:100 (*v*/*v*%). It was followed by continuous mixing for one hour. Then, the filler was left in the solution for the next 3 h and dried in an oven at 80 °C for 24 h. In this way, a filler modified with 3-isocyanatopropyltriethoxysilane was obtained. Such developed plum fillers (Figure 1) were used as a reinforcing filler in the synthesis of PUR composite foams.

### 2.2. Filler Characterization

**Figure 2:**
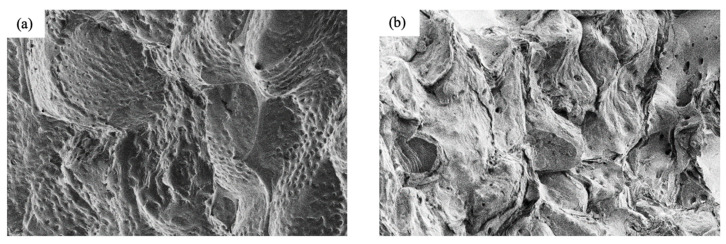
SEM images of plum fillers: (**a**) unmodified; (**b**) silanized.

**Figure 3:**
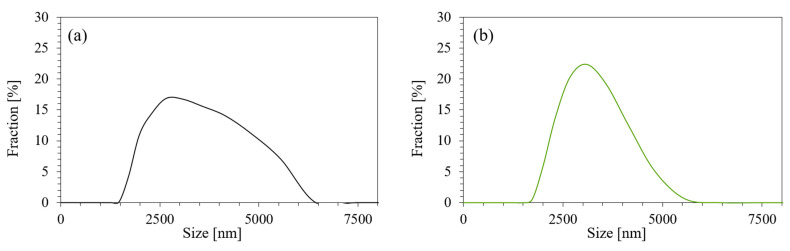
Distribution of particle size of (**a**) unmodified plum filler; (**b**) silanized plum filler.

The modification in the form of silanization influences the subsequent external morphology of the filler. As presented in Figure 2, the external morphology of nonfunctionalized plum filler is quite rough, while the external morphology of silanized plum filler is smoother. This is most likely due to the effect of attaching silanol groups to the plum filler surface. Figure 3 shows the particle size distribution of plum filler and silanized plum filler. Based on these data, it can be observed that the particles of the unmodified plum filler are more diverse and reach a little larger size. In the case of the silanized plum filler, the particles are more homogeneous, with a greater proportion of smaller sizes. This may be due to the additional grinding of the filler in a mortar immediately before the silanization process.

The fillers also influenced the dynamic viscosity of the polyol premixes. For both the plum filler and silanized plum filler, the viscosity of the polyol systems increased with increasing filler content.

It is worth noting that in the case of the unmodified plum filler, the effect on the increase in viscosity is greater. The dynamic viscosity increases from 680 mPa·s for PUR_REF to 2080 mPa·s and 1290 mPa·s for PUR_P5 and PUR_Si-P5, respectively. Moreover, based on the viscosity tests of the obtained polyol systems, it could be concluded that the resulting systems showed the properties of non-Newtonian shear thinning liquids.

### 2.3. PUR Composites Characterization

**Figure 4:**
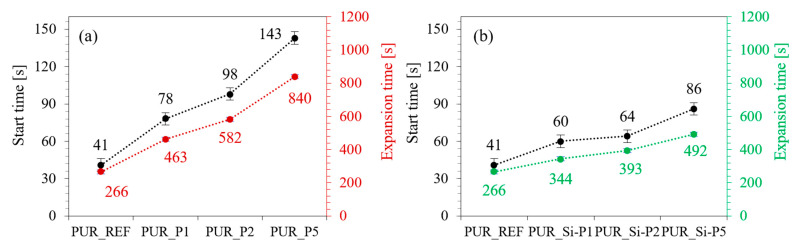
Results of the start time and expansion time of polyurethane foams with the addition of (**a**) unmodified plum filler (**b**) silanized plum filler.

As presented in Figure 4, the incorporation of plum fillers affects the processing times. When compared with PUR_REF, on the addition of unmodified plum filler, the start time increases by 90, 139, and 249%, while the expansion time increases by 74, 119, and 216% respectively, for PUR_P1, PUR_P2, and PUR_P5. When compared with PUR_REF, on the addition of silanized plum filler the start time increases by 46, 56, and 110%, while the expansion time increases by 29, 53, and 85%, respectively, for PUR_Si-P1, PUR_Si-P2, and PUR_Si-P5. According to the results presented in previous papers, the addition of natural, organic fillers may influence the stoichiometry of the reaction between hydroxyl groups and isocyanate of PUR systems. Differences between filler’s effect may come from the fact that PUR_Si-P fillers are modified with silane, which contains isocyanates groups, that can react with hydroxyl groups, which makes the reaction more efficient [60]. Moreover, the expansion of the pores is additionally reduced due to the higher viscosity of the system with the addition of the fillers, which explains the increased expansion time of the polyurethane foams modified with plum fillers. Similar trends were observed and reported in previous articles describing the preparation of PUR foams with the addition of other natural fillers [61].

**Figure 5:**
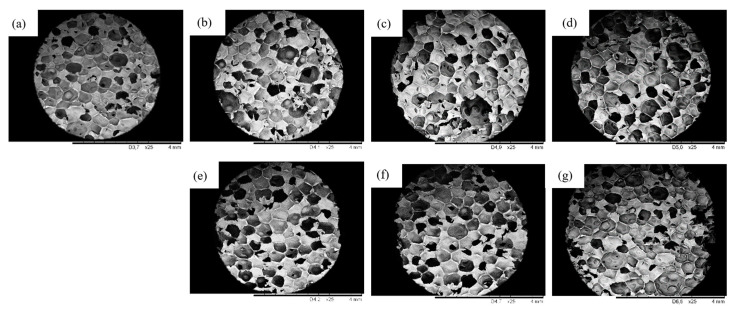
Cellular morphology of (**a**) PUR_REF, (**b**–**d**) PUR_P, and (**e**–**g**) PUR_Si-P.

The impact of plum filler addition on the cellular morphology of PUR composites was assessed by SEM. As presented in Figure 5, obtained PUR foams show a typical, polyhedral structure with a high content of closed cells. When plum fillers are added to the PUR systems, the average cell size is smaller. The average size of cell size decreases from 347 µm for PUR_REF to 335, 332, and 340 µm, respectively, for PUR_P1, PUR_P2, and PUR_P5 and to 330, 328 and 320 µm for PUR_Si-P1, PUR_Si-P2, and PUR_Si-P5. According to the SEM results, the application of plum fillers results in the formation of PUR composites with a higher content of open cells, which is related to a decrease in the closed-cell content. According to Table 1, it can be noticed that the number of closed cells decreased from 88.5% for PUR_REF to 87.8, 87.0, and 81.1%, respectively, for PUR_P1, PUR_P2, and PUR_P and to 87.9, 88.0, and 86.4%, respectively, for PUR_Si-P1, PUR_Si-P2, and PUR_Si-P5. The increased content of open cells may be due to the poor compatibility of the filler particles with the PUR matrix [62]. This can result in the collapse of the structure and the formation of open cells with a consequent weakening of the final structure of the obtained foams. Based on the obtained data, it can be observed that the incorporation of silanized filler caused a smaller decrease in the content of closed cells than the incorporation of unmodified filler. It may be related to the silanization process and greater compatibility of the modified filler with the polymer matrix [59,63]. The silanized plum filler particles can be successfully incorporated into the PUR structure, which slightly strengthens the resulting structure. Moreover, the silanized filler particles showed a smaller particle size distribution, which may also be reflected in the structure.

**Figure 6:**
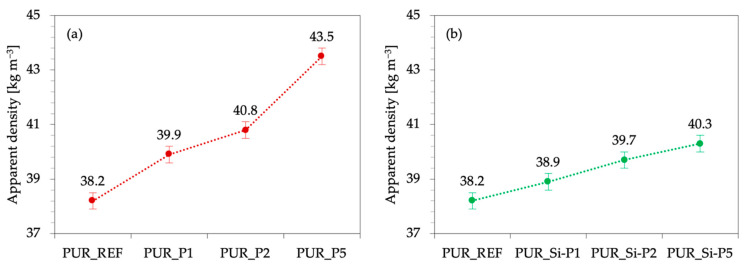
Results of apparent density determined for PUR composites modified with (**a**) plum filler and (**b**) silanized plum filler.

As presented in Figure 6, the incorporation of plum fillers also influenced the apparent density of the obtained composites. The value of apparent density increases from 38.2 kg m^−3^ for PUR_REF to 39.9, 40.8, and 43.5 kg m^−3^ for PUR_P1, PUR_P2, and PUR_P5 and to 38.9, 39.7, and 40.3 kg m^−3^ for PUR_Si-P1, PUR_Si-P2, and PUR_Si-P5, respectively. This may be due to the incorporation of plum fillers showing a higher density than the PUR matrix. Moreover, as shown in Table 1, the addition of plum fillers caused an increase in the dynamic viscosity of the polyurethane systems, which resulted in a difficult expansion of the bubbles and the formation of smaller cells of the structure. Therefore, modified foams show increased density.

**Figure 7:**
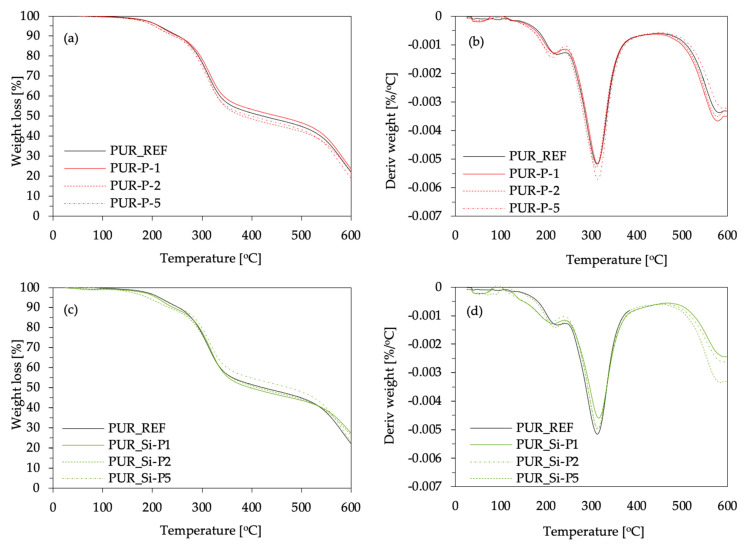
Thermogravimetric (TGA) and derivative thermogravimetry (DTG) results obtained for UR foams modified with (**a**,**b**) plum fillers and (**c**,**d**) silanized plum fillers.

Thermogravimetric analysis (TGA) and derivative thermogravimetry (DTG) analysis were used to assess the impact of plum filler and silanized plum filler on the thermal properties of the obtained polyurethane composite foams. During the measurement, the characteristic temperatures were determined based on the derivative thermogravimetry graph, as T_max1_, T_max2,_ and T_max3_. Moreover, char residues were measured at 600 °C. The obtained results are presented in Figure 7 and Table 2.

In the case of PUR foams, thermal degradation can be divided into three stages. The first stage of decomposition (T_max1_) starts at relatively low temperatures, between 210 and 230 °C, and is related to the thermal decomposition of low-molecular-weight compounds and the dissociation of the urethane bonds at a temperature between 150 and 330 °C [64,65]. The incorporation of unmodified and silanized plum fillers results in lower values of T_max1_, which may be related to the inhomogeneous dispersion of fillers in the matrix and change in cross-link density [66]. The second stage of thermal decomposition (T_max2_) occurs between 300 and 320 °C and refers to the thermal degradation of plum fillers and the thermal degradation of urea bonds in hard segments of the PUR structure [67]. At this stage of thermal decomposition, both the unmodified and silanized plum fillers slightly increased the thermal stability of the PUR composites.

The last step of thermal decomposition (T_max3_) refers to the degradation of the fragments generated during previous steps and occurs at a temperature between 570 and 590 °C. At this decomposition stage, a slight stabilizing effect of the plum fillers in terms of thermal stability is observed, with the best effect being observed for PUR_P5 for the addition of unmodified plum filler and PUR_Si-P1 for the addition of silanized plum filler. Furthermore, the thermal stability of polyurethane foams was confirmed by the char residue amount, measured at 600 °C. When analyzing char residue, it can be observed that the value decreases with the increase in the amount of filler for both unmodified and silanized filler. When comparing foams with the addition of unmodified plum filler with PUR_REF, the value increases from 21.9% to 23.5% for PUR_P1, stays at the same level (21.9%) for PUR_P2, and decreases to 18.9% for PUR_P5. When comparing foams with the addition of silanized plum filler with PUR_REF, an increase in the value to 27.6, 26.4, and 23.1% for PUR_Si-P1, PUR_Si-P2, and PUR_Si-P5, respectively, is observed. Higher carbon residue content may indicate the formation of more stable carbon layers, protecting the materials from further decomposition, and thus increasing thermal stability.

**Figure 8:**
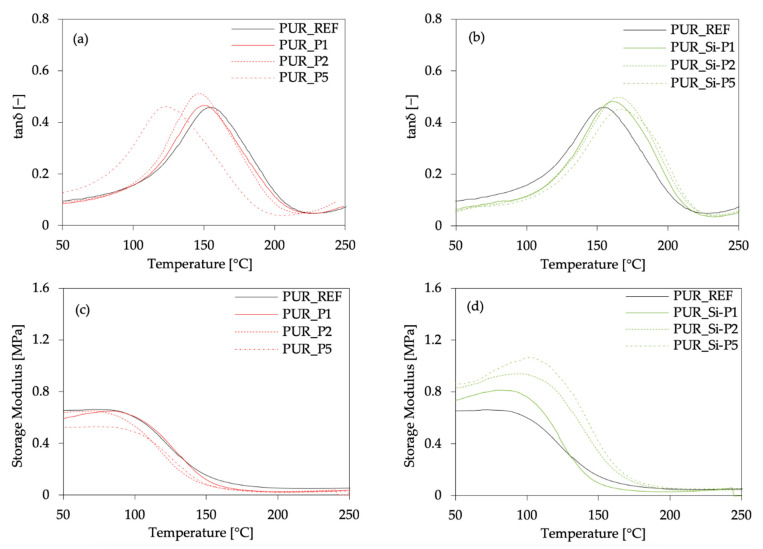
DMA results of PUR composites reinforced with unmodified and silanized plum fillers: (**a**,**b**) glass transition temperature (T_g_); (**c**,**d**) storage modulus (E′).

Dynamic mechanical analysis (DMA) was used to determine the reinforcing effect of plum fillers on the PUR foam composites. According to the results presented in Figure 8, the incorporation of unmodified plum filler affects the value of T_g_. PUR composites containing 1, 2, and 5 wt.% of unmodified plum filler exhibit lower values of T_g_ when compared with PUR_REF. Moreover, the value of T_g_ decreases from 156 to 125 °C as the content of unmodified plum filler increases from 1 to 5 wt.%, respectively. This effect may be connected with the poor interphase adhesion between filler surface and PUR matrix, which results in more porous structure creation and contributes to an increase in the mobility of the polymer chains. An opposite tendency is observed for PUR composites reinforced with silanized plum filler. When compared with PUR_REF, the values of T_g_ shift toward higher temperatures. The highest value of T_g_ exhibits PUR_Si-P5. Compared to PUR_REF, the value of T_g_ increases from 158 to 168 °C. These results are in agreement with the results of apparent density. As presented in Table 1, the values of apparent density of PUR composites containing silanized plum filler are somewhat higher, while the overall structure is more uniform with the greater number of closed cells compared to PUR composites reinforced with unmodified plum filler. This led to higher values of T_g_. The confirmation of the T_g_ results may be also found in the results of the storage modulus (E′). According to the results presented in Figure 8, the incorporation of each amount of unmodified plum filler decreases the value of E′ due to the more porous structure of PUR composites. The value of E′ increases after the incorporation of silanized plum filler. When compared with PUR_REF, the value of E′ increases by ~25, ~45, and ~62% after the addition of 1, 2, and 5 wt.% of silanized plum filler. This may be connected with the effective interactions between the silanized plum filler and the PUR matrix, which are supported by the functional groups on the surface of the plum filler. Previous studies have reported [68,69] that the functionalization of the fillers provides an effective filler–matrix interaction, which improves the reinforcement of the PUR composites. A similar explanation may be found in our study as well.

**Figure 9:**
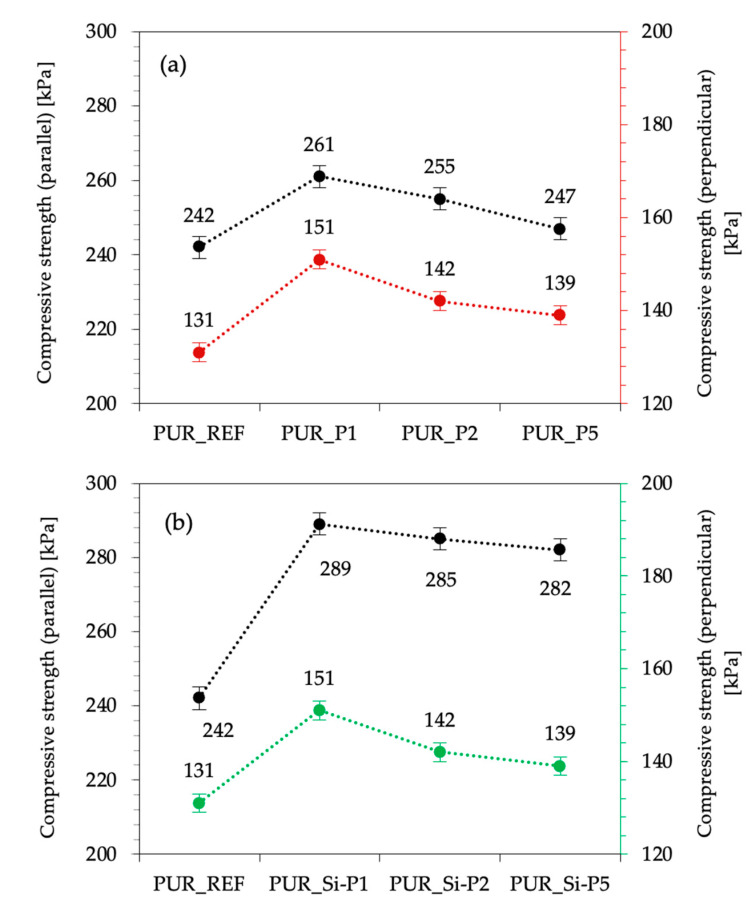
Effect of plum (**a**) and silanized plum (**b**) fillers’ content on the compressive strength of PUR foams.

The impact of plum fillers on mechanical properties was assessed by measuring the compressive strength at 10% deformation ($\sigma_{10\%}$) and flexural strength. As presented in Figure 9, the incorporation of unmodified plum filler and silanized plum filler affects the value of compressive strength. When compared with PUR_REF, the value of compressive strength, measured parallel to the direction of foam growth, increases by 8, 5, and 2% for PUR_P1, PUR_P2, and PUR_P5, respectively. An analogous trend occurs in the case of compression perpendicularly to the foam growth direction. When compared with PUR_REF, $\sigma_{10\%}$ increases by 15, 8, and 6% for PUR_P1, PUR_P2, and PUR_P5, respectively. In the case of modified plum filler, compared with PUR_REF, the value of compressive strength, measured parallel, increases by 19, 18, and 16% for PUR_Si-P1, PUR_Si-P2, and PUR_Si-P5, respectively. In the case of perpendicular compression, the increase in compressive strength is about 27, 23, and 20%, respectively, for PUR_Si-P1, PUR_Si-P2, and PUR_Si-P5, compared to the reference foam. Based on these data, it can be observed that the value of $\sigma_{10\%}$ decreases with the increasing amount of filler in both cases of unmodified and silanized plum filler. To avoid the apparent density impact in the mechanical properties of PUR foams, the specific compressive strength was determined as well. Moreover, for all polyurethane foams modified with silanized filler, the value of this parameter is higher (7.4 kPa/kg/m^3^ for PUR_Si-P1, 7.2 kPa/kg/m^3^ for PUR_Si-P2, and 7.0 kPa/kg/m^3^ for PUR_Si-P5) compared to the reference foam (6.3 kPa/kg/m^3^).

**Figure 10:**
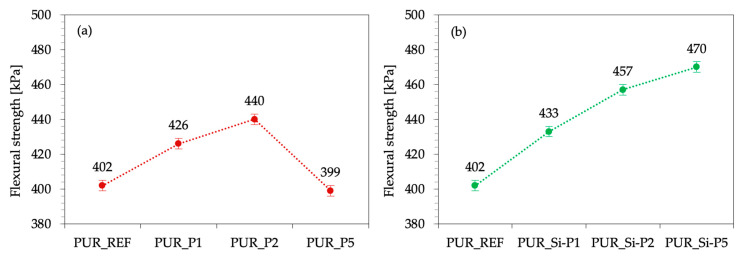
Effect of plum (**a**) and silanized plum (**b**) fillers’ content on the flexural strength of PUR foams.

The reinforcing effect of plum fillers was also verified by the results of flexural strength. As presented in Figure 10, when compared with PUR_REF, the addition 1 and 2 wt.% of the unmodified plum filler caused an increase in the flexural strength, but in the case of 5 wt.% of this filler, a significant decrease in the value of this parameter was observed. On the other hand, in the case of the silanized filler, a continuous tendency to strengthen the flexural strength is observed the greater the more filler is used.

Based on the obtained results, it can be concluded that the incorporation of plum fillers affects the mechanical properties of the obtained polyurethane foams, increasing their compressive strength (the best effect of PUR_Si-P1) and flexural strength (the best effect of PUR_Si-P5). This may be related to the possibility of filler particles incorporation into the PUR structure and the improvement of its parameters.

**Figure 11:**
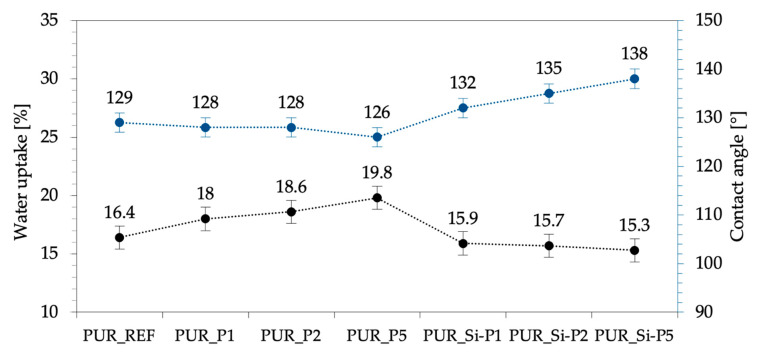
Selected properties of PUR foams: water uptake and contact angle results.

**Figure 12:**
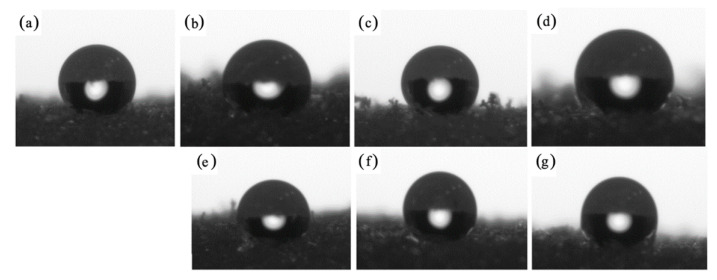
Images of the contact angle measured for (**a**) PUR_REF, (**b**–**d**) PUR_P, and (**e**–**g**) PUR_Si-P.

The hydrophobic nature of the obtained foams was evaluated using water uptake and contact angle analysis. Water absorption of porous materials depends mostly on their structure (the content of closed and open cells) and their character (hydrophobic or hydrophilic) [70,71]. As presented in Figure 11, the plum fillers used affected both the water uptake and the contact angle results. When compared with PUR_REF, the water uptake of foams increases by 10, 13, and 21% for PUR_P1, PUR_P2, and PUR_P5, respectively. This may be due to the higher content of open cells in the structure or the hydrophilic nature of the unmodified organic filler. When compared with PUR_REF, the water uptake of foams decreases by 3, 4, and 7%, respectively, for PUR_Si-P1, PUR_Si-P2, and PUR_Si-P5. This may indicate an increase in the hydrophobic character of the filler (through silanization), which reduces water absorption. The observed properties of the modified foams were confirmed by the contact angle test. When compared with PUR_REF, the contact angle decreases by 0.14, 0.77, and 1.19% for PUR_P1, PUR_P2, and PUR_P5, respectively. It is an almost imperceptible decrease of this value (within the error limit). However, in the case of foams with the addition of silanized plum filler, compared with PUR_REF, the contact angle increases by 2, 5, and 7% for PUR_Si-P1, PUR_Si-P2, and PUR_Si-P5, respectively. Increased values of the contact angle in the case of PUR_Si-P foams indicate reduced water absorption, which confirms the earlier assumptions and the results obtained during the water absorption test. Figure 12 shows the drops on the surface of the foams obtained during the contact angle test.

## 3. Materials and Methods 

### 3.1. Materials

- Polyether polyol with a brand name of Stepanpol PS-2352, purchased from Stepan Company (Northfield, IL, USA);
- Polymeric diphenylmethane diisocyanate with a brand name of Purocyn B, purchased from Purinova Company (Bydgoszcz, Poland);
- Kosmos 75 (potassium octoate) and Kosmos 33 (potassium acetate), purchased from Evonik Industry (Essen, Germany);
- Tegostab B8513 (silicone-based surfactant), purchased from Evonik Industry (Essen, Germany);
- Pentane, cyclopentane, purchased from Sigma-Aldrich Corporation (Saint Louis, MO, USA);
- 3-isocyanatopropyltriethoxysilane, provided by Sigma-Aldrich Corporation (Saint Louis, MO, USA);
- Ground plum stones were supplied by a local company from Poland.

### 3.2. Methods and Instruments

The average size of plum filler particles was determined with the dynamic light scattering (DLS) method, using a Zetasizer NanoS90 instrument (Malvern Instruments Ltd., Malvern, UK)

The morphology of foams and cell size distribution were analyzed on the basis of the cellular structure pictures of the obtained foams, taken using the JEOL JSM-5500 LV scanning electron microscope (JEOL LTD, Akishima, Tokyo, Japan). The microscopic analysis was carried out in a high-vacuum mode and at the accelerating voltage of 10 kV. Foam samples were scanned in a parallel direction to the foam growth. The average pore diameters and pore size distribution were measured using ImageJ software (Media Cybernetics Inc., Rockville, MD, USA).

The apparent density of analyzed foams was determined in accordance with the standard ASTM D1622 (which is equivalent to ISO 845) as the ratio of sample weight to its volume. The density was measured on five samples of each foam and expressed as an average.

The thermal stability of analyzed foams was determined using the Mettler Toledo thermogravimetric analyzer TGA/DSC1. The thermal decomposition study was conducted in an inert gas atmosphere (flow 50 mL min^−1^) and in the temperature range between 25 and 600 °C (with a heating rate of 10 °C min^−1^). The experiment included analysis of the mass change as a function of temperature during thermal decomposition of the PU foams. The initial temperatures of the following stages of decomposition were noticed and designated as T_5%_, T_10%_, and T_50%_. These values of temperature correspond to the weight percentage loss.

Dynamic mechanical analysis (DMA) was determined using an ARES rheometer (TA Instruments, New Castle, DE, USA). Measurements were carried out in the temperature range of 20–250 °C at a heating rate of 10 °C min^−1^ using a frequency of 1 Hz and applied deformation at 0.1%.

The compressive strength ($\sigma_{10\%}$) of analyzed foams was determined in accordance with the standard ASTM D1621 (which is equivalent to ISO 844). The analysis was carried out using the Zwick Z100 Testing Machine (Zwick/Roell Group, Ulm, Germany) with a load cell of 2 kN and a speed of 2 mm min^−1^. Compression strength was determined as a ratio of the load causing 10% deformation of the cross-section of samples both perpendicular and parallel to the square surface. Compressive strength was tested on at least five samples in series and expressed as an average.

The three-point bending test was carried out in accordance with the standard ASTM D7264 (which is equivalent to ISO 178) using the Zwick Z100 Testing Machine (Zwick/Roell Group, Germany). Foam samples were bent with a speed of 2 mm min^−1^. For each foam series, at least five measurements were made. The obtained flexural stress at the break results for each sample was expressed as a mean value and averaged.

Surface hydrophobicity was examined by contact angle measurements using the sessile drop method. The examination was performed using a manual contact angle goniometer with an OS-45D optical system (Oscar, Taiwan) to capture the shape of liquid on the solid surface. A water drop of 1 µL was deposited using a micrometer syringe fitted with a stainless-steel needle onto the flat surface neatly cut out from the inside of the foam. The contact angles were measured at least ten times on each sample and averaged.

Water absorption of foams was measured according to the standard ASTM D2842 (which is equivalent to ISO 2896). Tested samples were dried at 80 °C for 1 h and weighed. Subsequently, samples were immersed in distilled water for 24 h to the depth of 1 cm. Then, samples were removed from the water, held vertically for 10 s, and dried between dry filter paper (Fisher Scientific, Waltham, MA, USA) at 10 s and weighed again. Water absorption was measured on five samples of each foam and averaged.

### 3.3. Polyurethane Composite Synthesis

**Figure 13:**
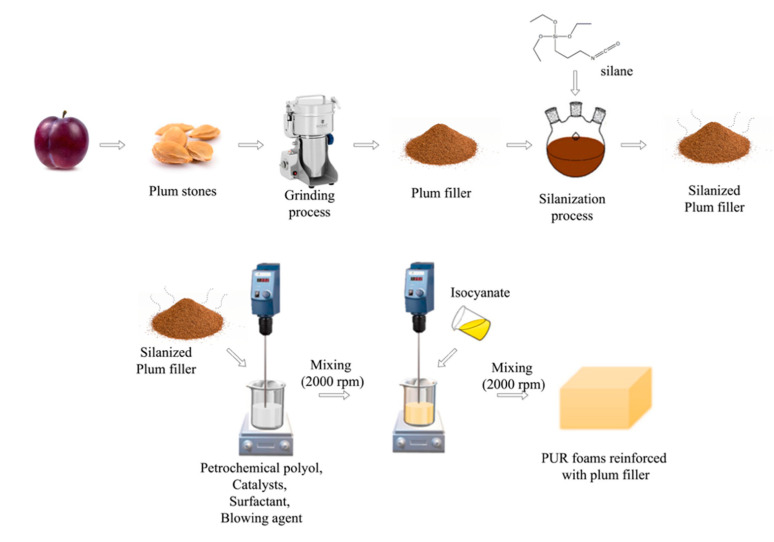
Schematic procedure of the synthesis of polyurethane foams reinforced with unmodified and silanized plum filler.

The calculated amounts of polyol, filler, catalysts, blowing agent, and surfactant were placed in a container and mixed thoroughly (2000 rpm, 60 s). Then, the isocyanate compound was added into the container with thorough stirring (2000 rpm, 30 s). In line with the supplier information, the isocyanate was mixed in a 100:160 ratio (polyol to isocyanate) to ensure the complete reaction between the hydroxyl and isocyanate groups. The polyurethane composites were cured for 48 h at room temperature. All compositions of the prepared composites are listed in Table 3. The scheme of the procedure for the synthesis of polyurethane foams is presented in Figure 13.

## 4. Conclusions

Polyurethane foams were successfully reinforced using 1, 2, and 5 wt.% of ground plum stones (P) and silanized ground plum stones (Si-P). The addition of these fillers into polyol systems increased their viscosity and prolonged the characteristic times of PUR obtaining. The analysis of the impact of fillers indicates that the incorporation of 1–5 wt.% of plum filler and silanized plum filler affects the cellular structure of PUR composites and their further thermal, mechanical, and application properties. SEM images show reduced cell size but higher open-cell content in the case of foams with the addition of the fillers. Moreover, the results showed that compared to PUR_REF, the addition of plum filler and silanized plum filler leads to the obtaining of PUR composite foams with improved physicomechanical, thermal, and application properties. The best results were obtained for PUR foams modified with silanized plum filler. For example, the incorporation of 1 wt.% of Si-P filler could provide polyurethane foams with better compressive strength (improvement by ~19%). The incorporation of 5 wt.% of Si-P filler could provide PUR composites with improved flexural strength (improvement by ~17%). Moreover, such foams have a higher contact angle value (increase by ~7%) and reduced water uptake (reduction by ~7%). Furthermore, polyurethane foams reinforced with silanized plum filler showed improved thermal and thermomechanical stabilities compared to polyurethane foams with the addition of unmodified plum filler. The presented results confirm that the use of P and Si-P as fillers in polyurethane composite foams is a good path for the application of natural waste converted into a useful resource.

## Figures and Tables

**Table 1:** Rheological and structural properties of polyurethane foams.

	PUR_REF	PUR_P1	PUR_P2	PUR_P5	PUR_Si-P1	PUR_Si-P2	PUR_Si-P5
Dynamic viscosity at 10 rpm (mPa·s)	680 ± 7	1083 ± 6	1577 ± 7	2080 ± 5	852 ± 6	989 ± 8	1290 ± 5
Cell size (µm)	347 ± 2	335 ± 3	332 ± 2	340 ± 4	330 ± 3	328 ± 5	320 ± 4
Closed-cell content (%)	88.5 ± 0.2	87.8 ± 0.3	87.0 ± 0.4	81.1 ± 0.8	87.9 ± 0.7	88.0 ± 0.6	86.4 ± 0.5
Apparent density (kg m^−3^)	38.2 ± 0.7	39.9 ± 0.5	40.8 ± 0.8	43.5 ± 0.6	38.9 ± 0.4	39.7 ± 0.7	40.3 ± 0.9

**Table 2:** Results of the thermal stability of PUR foams.

Sample	T_max_ (°C)			Char Residue (wt.%) at 600 °C
	1st Stage	2nd Stage	3rd Stage	
PUR_REF	226	311	578	21.9
PUR_P1	220	314	581	23.5
PUR_P2	215	311	579	21.9
PUR_P5	214	314	588	18.9
PUR_Si-P1	215	315	589	27.6
PUR_Si-P2	221	314	585	26.4
PUR_Si-P5	212	313	584	23.1

**Table 3:** Composition of polyurethane foams.

Component	PUR_REF	PUR_P1	PUR_P2	PUR_P5	PUR_Si-P1	PUR_Si-P2	PUR_Si-P5
	Parts by Weight (wt.%)						
STEPANPOL PS-2352	100	100	100	100	100	100	100
PUROCYN B	160	160	160	160	160	160	160
Kosmos 75	6	6	6	6	6	6	6
Kosmos 33	0.8	0.8	0.8	0.8	0.8	0.8	0.8
Tegostab B8513	2.5	2.5	2.5	2.5	2.5	2.5	2.5
Water	0.5	0.5	0.5	0.5	0.5	0.5	0.5
Pentane/cyclopentane	11	11	11	11	11	11	11
Plum filler	-	1	2	5	-	-	-
Silanized Plum filler	-	-	-	-	1	2	5

## Data Availability

Data sharing not applicable.
